# An analysis of Euroqol EQ-5D and Manchester Oxford Foot Questionnaire scores six months following podiatric surgery

**DOI:** 10.1186/1757-1146-5-17

**Published:** 2012-07-09

**Authors:** Anthony J Maher, Timothy E Kilmartin

**Affiliations:** 1 Department of Podiatric Surgery, County Health Partnerships, Park House Health and Social Care Centre, Burton Road, Carlton, Nottingham, NG4 3DQ, UK; 2 Department of Podiatric Surgery, Derbyshire Community Health Service, Ilkeston Community Hospital, Heanor Road, Ilkeston, Derbyshire, DE7 8LN, UK

## Abstract

**Background:**

In the United Kingdom patient-reported outcome measures (PROMS) have been adopted as a key measure of foot surgery outcomes. The intention of this study was to evaluate the responsiveness of a regional outcome measure; the Manchester Oxford Foot Questionnaire (MOXFQ) and a generic measure; the EuroQol EQ-5D, in the context of day care Podiatric Surgery.

**Methods:**

A prospective audit of 375 consecutive day care surgical admissions was undertaken. All patients attending for surgery, who agreed to participate, were included. Pre operation patients completed the MOXFQ and the EQ-5D. Both questionnaires were completed again at 6 months post operation. Additional data was collected on patient demographics, surgical procedures and complications.

**Results:**

Few complications were encountered and most patients (84%) returned for a final review 6 months post operation. Mean MOXFQ scores improved for each domain: pain; 51.7 pre-operation, reduced to 16.5 post-operation, walking; 50.2 reduced to 14.1 and social interaction; 45.7 reduced to 10.6. The minimal clinically important differences (MCID) estimates for the pain domain were exceeded by 82.6% of patients, while 74.8% exceeded the MCID for walking and 68.5% exceeded the MCID for social interaction. A small number of patients (2.9%) deteriorated across all three MOXFQ domains.

The EQ-5D Index, summary of health related quality of life, improved from 0.66 pre-operation to 0.86 post operation. The EQ-5D index MCID was exceeded by 79.2% of patients. Index scores deteriorated for 1.8% of patients following surgery. Effect sizes measured following surgery were largest for the MOXFQ domains: Walking; 1.39, Pain; 1.52 and Social Interaction: 1.39. The EQ-5D index effect size was 0.83. The EQ-5D visual analogue scale (VAS) was not influenced by surgery.

**Conclusion:**

Both the MOXFQ and EQ-5D index (but not the VAS) appear sensitive to changes in health status at 6 months following elective foot surgery. Both instruments were particularly responsive to changes in pain, mobility and activity or social interaction following treatment. The MOXFQ was developed specifically for foot surgery and as such appears to be the more sensitive instrument. However the generic EQ-5D may allow comparison of general health states in the wider health community. Both instruments when used together appear well suited to the measurement of change in perceived health status following foot surgery.

## Background

As with medicine and healthcare in general, there has been in recent years, a drive within foot surgery to adopt a more robust approach to measuring outcomes. Historically there had been a reliance on simple audits of clinical outcomes and patient satisfaction [1,2]. However, more recently foot specialists in the United Kingdom have adopted patient reported outcome measures (PROMs) as a key measure of the outcome of an intervention [3-7].

The choice of outcome measures available in foot and ankle surgery is extensive though It has long been thought that within a specialist field such as foot surgery, it is more appropriate to use specific regional or anatomic measures of outcome such as the Manchester Oxford Foot Questionnaire (MOXFQ) [8] rather than a generic measure such as the EQ-5D. Generic questionnaires may be less responsive to the subtle changes that can occur in foot health (following intervention), which in-turn have an impact on health status or health related quality of life [9]. Responsiveness is a term which refers to the ability of an instrument to detect important changes in health status following treatment (such as surgery) or over a period of time, it is not standardised for a given instrument and may be influenced by external patient factors [10]. Effect sizes (ES) may be used as a statistical measure of responsiveness. An ES represents the magnitude of change identified by an instrument in a unitless way that allows direct comparisons to be made between instruments [10,11]. This paper will evaluate both the MOXFQ and EQ-5D in the context of elective day care foot surgery.

The MOXFQ is a recently established instrument developed for the measurement of outcomes in foot and ankle surgery [8,12,13]. The MOXFQ (MOXFQ© 2011, Isis Innovation Ltd) is a PROM which was originally developed using interviews with patients having hallux valgus surgery and drawing on the Manchester Foot Pain & Disability Questionnaire (MFPDQ) [14], as a template. MFPDQ development had involved interviews with community based patients with a range of foot and ankle conditions. Changes were made to both item content & response categories, to produce a measure (the MOXFQ) more suitable for the surgical context, including the addition of items concerning night and day-time pain severity levels. The modified questionnaire was subsequently tested for dimensionality, internal reliability, responsiveness, construct validity, and divergent/convergent validity [8,15]. Further testing has confirmed the validity of the MOXFQ in the context of hallux disorders, digital, midfoot, hindfoot and ankle pathology [12]. The instrument comprises 16 questions each with a 5 point Likert scale answer. The questions are divided between three domains; walking & standing; pain; social interaction. For each domain, summed scores are converted to a 0-100 scale where high scores denote most severe symptoms or problems.

The MOXFQ is growing in popularity amongst specialists in foot surgery across Europe [7,16-18]. The Society of Chiropodists and Podiatrists in the United Kingdom have now adopted the MOXFQ as the standard instrument for assessing patient reported outcomes following foot surgery [1].

The EQ-5D was developed in 1987 as a generic measure of health status [19]. It was tested for construct validity in 1993 and test re-test reliability in 1994. [19-22]. Data is available for the EQ-5D across 15 geographic populations and for many specific patient groups, to date the instrument has been used in over 500 studies [23,24]. The EQ-5D is a 5 item questionnaire with only three possible response options (1 = no problem, 2 = moderate problem, 3 = severe problem). The five EQ-5D questions cover; mobility; self care; usual activities; pain and discomfort; anxiety and depression. Alongside the EQ-5D a VAS scale (EQ-5D Health status thermometer) asks patients for their subjective opinion of their health status [23]. Additionally the EQ-5D data can be manipulated to generate a health index with a score range of –1.0 to 1.0, where 1.0 equals best possible health state and –1.0 equates to a state worse than death [25].

Despite its wide spread use in medicine and epidemiology, there are very few references to the EQ-5D within the field of foot surgery [18,26,27]. At face value there seems to be some overlap in the EQ-5D and MOXFQ domains. Both instruments measure pain although the EQ-5D will measure pain occurring anywhere in the body, as opposed to the foot. Both also measure mobility and activity, while the EQ-5D additionally measures anxiety and ability to self care.

Minimal clinically important differences (MCID) have been estimated for both instruments [15,28]. The MCID is an anchor-based estimate (using a transition item as the anchor) of the smallest amount of change (usually improvement) in a health status score that patients can generally detect. This compares with small clinically irrelevant changes in health status that may be statistically significant despite being of ‘no value’ to the patient. It is also possible for a clinically important change in status to not reach statistical significance, particularly if the sample size is small.

Beyond their own work on hallux valgus outcomes, the authors of the current paper are not aware of any other published reports of podiatric surgery in the United Kingdom which have incorporated the EQ-5D as a generic measure of health status in addition to a specific regional measure [7].

The intention of this study was therefore to evaluate the responsiveness of a regional outcome measure; the MOXFQ and a generic measure; the EQ-5D, six months following elective day care foot surgery performed by podiatric surgeons. A null hypothesis was formulated stating that a foot specific measure would be no more responsive than a generic measure of health related quality of life.

## Methods

Approval was sought, from a local governance and audit committee, to implement two PROMs instruments; the EQ-5D (Additional file 1) and the MOXFQ. These were to be used alongside the existing surgical audit framework for all patients undergoing elective podiatric surgery at Ilkeston Community Hospital. Podiatrists working with the surgical team recruited patients during routine pre operative assessment clinics. All patients attending for elective day care podiatric surgery between May and December 2009 were given the opportunity to complete both the EQ-5D and MOXFQ pre and post operation. Beyond attending for podiatric surgery, there were no specific inclusion or exclusion criteria. All diagnoses encountered and surgical techniques employed during the study period were included. All surgery was performed as a day case on a single foot (i.e. unilateral); there were no bilateral procedures in the study period.

The existing audit framework comprised of clinical observations and retrospective data collection at 6 months post intervention. All patients in the department were routinely recalled to clinic at 6 months post operation for clinical evaluation with the intention being to discharge at that point if symptom free. Data relating to demographics, surgical activity and complications was entered onto a Microsoft Access database by a Podiatrist working with the surgical team.

### Instruments

Both the EQ-5D and MOXFQ were completed by patients pre operation, on the day surgery ward and again at 6 months post operation in the out-patients waiting room. No questionnaires were completed by post or electronically. If patients failed to attend a 6 month review appointment, no further attempt was made to collect PROM data.

Both sets of PROM data were entered onto a Microsoft Excel spreadsheet. The EQ-5D index calculations were taken from established UK population data using the time trade off method [23,25].

### Statistics

Analysis was undertaken with either the analyse-it statistical package version 2.2 or Microsoft Excel as appropriate [29]. Descriptive statistics (mean and standard deviation) are presented for all interval or ordinal data.

Effect sizes (ES) were chosen as a key assessment of responsiveness for both instruments. Effect sizes allow for instruments with differing measurement scales to be analysed side by side with respect to their ability to detect a change in scores [15]. The ES was assessed for the EQ-5D items, EQ-5D index, EQ-VAS and the MOXFQ domains. Effect sizes were calculated using the formula; $ES=\frac{M1-M2}{SD}$, where *M1* is the pre operative mean and *M2* the post operative mean score and *SD* is the standard deviation of the mean pre operative score. ES values of 0.2, 0.5 and 0.8 are typically regarded as indicating small, medium and large degrees of change, respectively [11]. An ES of 0.2 to <0.5 was considered small, 0.5 to <0.8 considered moderate and >0.8 considered large. An ES of 1.0 is equivalent to a change of one SD in the sample score. For the purposes of the current study, an ES of 0.5 or greater was considered clinically important.

The Shapiro-Wilk test of normality was applied to all pre and post operative data. Normally distributed data was assessed for significance with the Student’s *T* test. Non normally distributed data was analysed with the Wilcoxon Test. P values are presented throughout with a value of <0.05 taken to represent statistical significance.

## Results

### Demographics and complications

Between the 18^th^ May 2009 and the 9^th^ December 2010, 375 patients attended for day care foot surgery. A total of 60 patients (16%) were lost to follow up having failed to attend for a final clinical review and so were excluded from further analysis. Surgical data was available for 315 patients while PROMs data was available for 304 patients (96.5%). A summary of the data collected is presented in Table 1. A broad range of podiatric diagnoses were encountered and an array of surgical procedures was applied to these diagnoses, the procedures are summarised in Table 2. Complications were recorded throughout the post operative period and are recorded in Table 3.

**Table 1:** Study period, Recruitment and Patient Demographics

**Total Population (Patients)**	375	Lost to follow up	60
Patients Included in study	315		
Patients with surgical data	315		
Patients with pre/post MOXFQ	304		
Patients with pre/post EQ-5D	279		
**Study Period**			
Start	18/05/2009	Finish	26/10/2010
Mean follow up (wks)	26.9	(SD 4.3)	
Min follow up	17	Max followup	79
**Age and sex**			
Min	17	Max	88
Mean	58.5	(SD12.3)	
Female	85%	Male	15%
**ASA Grade**			
1	43.8%		
2	53.3%		
3	2.9%		

**Table 2:** Summary of Surgical Intervention

**Total Procedures**	**409**
Pt's receiving more than one procedure	81
Mean procedures per patient	1.298
**Specific Surgical Procedures (elective day case)**	
Hallx valgus repair (scarf-Akins procedure)	156
Digital surgery	132
Hallux Rigidus repair (osteotomy, arthroplasty, arthrodesis)	29
Skin, nail or Soft tissue	22
Lesser metatarsal (osteotomy, condylectomy)	19
Neurectomy	19
Cheilectomy	13
mid foot arthrodesis	5
Fixation removal	5
amputation (Digital; whole or partial)	3
sesamoid reduction	3
Hallux Varus repair (Reverse scarf procedure)	2
Sub Talar joint Arthroeresis	1

**Table 3:** Surgical Complications recorded post operation

**Complication**	**Count**	**Percentage**
Incision line healing	16	5.08
Recurrence	16	5.08
Transfer metatarsalgia	10	3.17
Infection suspected	7	2.22
Pain around site of surgery	7	2.22
Other	6	1.90
Swelling (abnormal)	5	1.59
Joint pain and stiffness	5	1.59
Scar line	4	1.27
Medication side effect	4	1.27
Infection proven	2	0.63
DVT (confirmed)	2	0.63
Sensory loss (small)	2	0.63
D.V.T (suspected)	1	0.32
**Total**	87	

### MOXFQ

MOXFQ data was available for 304 patients. Table 4 summaries the MOXFQ outcomes for the cohort. Dawson et al. previously estimated minimal clinically important differences (MCID) for each of the three domains [15]. These are 16 for walking, 12 for pain and 24 for social interaction. The current cohort exceeded the above score changes and so Podiatric Surgery was considered to have had a positive impact on the cohorts health related quality of life as defined by the MOXFQ. It was noted that it is possible for a patient to improve or deteriorate independently across the three domains of walking, pain and social interaction. We therefore looked at the numbers of patients improving or deteriorating across each individual domain (Table 5). Podiatric surgery had the greatest impact on the pain domain and the least impact on the social interaction domain.

**Table 4:** Summary of mean MOXFQ domain scores and mean EQ-5D scores pre and post treatment

		**Pre**		**Post**		**Score change**		Sig*	ES*
		**Mean**	**SD**	**Mean**	**SD**	**Mean**	**SD**		
**MOXFQ**	Walking	50.2	26.0	14.1	20.5	36.1	27.9	<0.0001	1.39
	Pain	51.7	23.1	16.5	19.0	35.2	25.9	<0.0001	1.52
	Social	45.7	25.3	10.6	17.4	35.1	27.3	<0.0001	1.39
**EQ-5D**	Index	0.7	0.2	0.9	0.2	0.2	0.1	<0.0001	0.83
**EQ-5D**	VAS	8.1	1.6	8.2	1.7	0.1	3.2	0.3050	0.04

*Sig; significance. *ES; Effect Size. SD; Standard deviation.

**Table 5:** Analysis of EQ-5D and MOXFQ minimal clinically important differences following treatment

	**EQ-5D index**		**MOXFQ Domains**					
			**Pain**		**Walking**		**Social**	
	**Count**	**%**	**Count**	**%**	**Count**	**%**	**Count**	**%**
**Deteriorated**	5.0	1.8	18.0	5.9	18.0	5.9	23.0	7.5
**no change**	53.0	19.0	35.0	11.5	59.0	19.3	73.0	23.9
**Improved(Exceeded MCID)**	221.0	79.2	252.0	82.6	228.0	74.8	209.0	68.5
**n.**	279.0	100.0	305.0	100.0	305.0	100.0	305.0	100.0

One hundred and seventy six patients (57.7%) had score improvements which exceeded the MCID for all three domains. Eight patients improved but failed to reach the MCID across any of the domains. Nine patients (2.9%) deteriorated across all three domains. This leaves 36.7% of patients whose MOXFQ scores either did not change or improved or deteriorated variably across the three domains.

### EQ-5D

Pre and post operative EQ-5D data was available for 279 patients. There were improvements across all but the self-care domain (Table 6). However, only 11 patients reported self care problems pre operation and the same group remained unchanged following surgery. The EQ-5D scores were converted to the EQ-5D index using the UK TTO method [23], Table 4 summarises the EQ-5D index data. Reference levels for the MCID have been described by Walters and Brazier [28]. Five patients deteriorated (1.8%) while 79.2% exceeded the MCID (Table 5). Despite the significant change in index scores post intervention, there was little change in the EQ-VAS post intervention suggesting that podiatric surgery patients actually perceived their overall ‘general health’ as representing something different (and in this sample generally as ‘good’) from any specific problems relating to pain and mobility (Table 4).

**Table 6:** EQ-5D items

	**Mobility**	**Self-care**	**Activities**	**Pain**	**Anxiety**
Pre	156	11	136	249	70
Post	67	11	47	104	38
Effect Size	0.63	0.00	0.61	1.25	0.24

The table lists the number of Patients reporting problems for each EQ-5D item pre and post surgery (with effect size).

### Effect sizes

In order to assess the magnitude of the change across each domain, Effect Sizes (ES) were measured and are presented in Tables 4 and 6. The ES were generally larger for the MOXFQ than for the EQ-5D. The largest ES for both instruments related to pain; 1.524 for the MOXFQ and; 1.254 for the EQ-5D. The effect sizes were also large for the MOXFQ walking/standing and social interaction domains. Moderate ES were found with the EQ-5D mobility and activities items. There was a small ES for the EQ-5D anxiety item. The EQ-5D index also demonstrated a large ES (0.83).

## Discussion

This paper has attempted to evaluate the responsiveness of two discrete health related quality of life instruments. Each has their own set of benefits and disadvantages. The MOXFQ is a very sensitive instrument able to detect subtle post operative changes in pain, mobility and social interaction. The effect sizes were larger for the MOXFQ which may be a consequence of the fact that this instrument was developed specifically to measure foot surgery outcomes and so increased sensitivity to change would be expected. It is interesting to note that despite the overall positive improvement in mean scores and the tendency to exceed the MCID across all three domains, only 57.7% of patients achieved a clinically significant improvement in each of the three domains. This suggests that there is considerable variability in individual needs, response to surgery and patient expectations. Unrealistic expectations are a recognised cause of poor outcomes and in the context of foot surgery this may manifest as a wish to be able to wear fashionable shoes or what the patient considers ‘normal’ shoes post treatment [30-35]. We might speculate that failure to meet such expectations and the associated dissatisfaction may negatively affect the MOXFQ social interaction score.

The MOXFQ pain domain was most reliably improved by surgery with 82.6% of patients reporting lower pain scores following intervention. Patients attending for podiatric surgery typically expect pain relief, followed by improved mobility and shoe fitting [30,36].

Very few patients suffered a deterioration of health status following intervention, with rates ranging from 5.9 – 7.5% across the domains. With reference to the MOXFQ, only 2.9% of patients deteriorated across all three domains. We did not investigate why these patients deteriorated post intervention and whether or not there was an association with complications, satisfaction or delayed recovery.

As with the MOXFQ, the EQ-5D appeared sensitive to changes in health status following intervention with improvements in item scores for pain, mobility, activity, and depression/anxiety. Self care scores did not change significantly following intervention but this was expected given that the majority of foot surgery patients are fully ambulatory. Few patients complained of anxiety or depression, though there was a small improvement in anxiety scores post intervention. This change may have been a consequence of asking patients to complete the pre operative questionnaire immediately prior to their surgery, when anxiety is likely to be a prominent feature.

EQ-5D Pain, Mobility and Activity items demonstrated the greatest score change following intervention. Similarly, the MOXFQ demonstrated improvement in pain, walking/standing and social interaction scores. Based on the current study, it could be assumed that anxiety and self care as measured by the EQ-5D are not significant concerns for patients undergoing ambulatory foot surgery. Macran et al. investigating health status in patients attending for routine Podiatry treatment similarly concluded that anxiety/depression and self care concerns are not an important characteristic of patients with foot disorders [37].

The combined responses to each item of the EQ-5D generates an index which does appear to be a useful tool for the assessment of health related quality of life following foot surgery in the short-term. An advantage of utilising the EQ-5D index is that it can be used as a comparative measure of health status for a variety of chronic conditions. The adoption of the EQ-5D index scores creates a ‘level playing field’ allowing an unbiased assessment of health need and outcomes. The mean EQ-5D index score for patients prior to Podiatric Surgery was 0.66. An age matched sample of the UK population has previously reported index scores of 0.8 [23]. By way of comparison to the current study, patient groups with scores of between 0.62 and 0.73 include sufferers of lower back pain, Parkinson’s disease, Rheumatoid Arthritis functional class 1 and patients at 6 months following a stroke [38-40]. It is intriguing to note that following foot surgery, the mean index score rises to 0.86, exceeding that of the age matched population sample.

This finding suggests that patients attending for podiatric surgery are, for the most part in reasonable general health in respect of systemic diseases but are specifically hampered by local (foot) pathology which causes pain and impedes mobility. The majority of patients in the current study (97.1%) were systemically well or suffered only minor ailments controlled with medication. The EQ-5D VAS is a subjective assessment of the patient’s health state using a ‘health thermometer’. Pre and post operation the VAS scores remain relatively high (8.12 and 8.18 respectively). Suggesting that despite foot pain and mobility difficulties, patients’ actually considered their health state to be good.

Effect sizes standardise different scoring systems which allows researchers to directly compare the magnitude of a change detected by two distinct instruments. Perhaps as would be expected, the effect sizes for the MOXFQ are considerably larger than those for the similar domains of the EQ-5D. The moderate effect sizes demonstrated by the EQ-5D for mobility and activity, and the large effect size for pain may offer complementary evidence for the impact of foot pathology and subsequent surgery on a patient’s systemic health status.

This paper was based on a clinical audit and presents the PROMs scores for two discrete instruments. Both instruments were completed by the majority of patients. There was no attempt at randomisation and all surgical procedures were included. In essence this paper represents a consecutive case series of patients undergoing a typical range of elective foot surgery procedures. As such we have to be guarded in the conclusions we may draw. Where possible, variables were controlled. Surgery was undertaken by one of three Podiatric Surgeons, all procedures were performed in a day surgery setting and all patients underwent a standard post operative rehabilitation program.

There are a couple of important weaknesses in the design and data capture of the current study. First; 60 patients or 16% of the total cohort failed to return for a clinical review at 6 months post operation. We do not know how the missing patients would have influenced the study data and the conclusions we have drawn. As a consequence there is a possible response bias. The overall trend in the current study was towards improved HRQOL following treatment but the missing cohort may well have deteriorated.

A second important consideration is that we did not examine why certain patients failed to improve or actually suffered deterioration in health status post treatment. The current study cannot for example, illicit whether co-morbidities or post operative complications influence post operative HRQOL scores although the majority of patients were relatively healthy. We did not control for medical conditions which may have arisen subsequent to surgery which may have influenced post operative scores.

The follow up period was set at 6 months, which was a relatively short period particularly for reconstructive foot surgery where the patient may continue to improve over 12 months. Assessing patients relatively early in their post operative recovery may not provide a realistic measure of the final outcome.

There would be value in further research to investigate the relationship between poor HRQOL scores post treatment and the incidence of complications, co-morbidities or unrealistic patient expectations.

## Conclusion

Measuring health status adds an additional dimension to the assessment of outcomes in Podiatric Surgery. The choice of outcome measure can often seem overwhelming. The ideal instrument will attempt to quantify the outcome of surgery in terms relevant not only to the care providers but also to the wider health community and the patients themselves.

Both the MOXFQ and EQ-5D appear sensitive to changes in health status following foot surgery. Both instruments were particularly sensitive to changes in pain, mobility and activity or interaction following treatment. The MOXFQ was developed specifically for foot surgery and as such appears to be the more sensitive instrument. However the generic EQ-5D may allow better comparison of health states in the wider health community. Both instruments when used together appear well suited to the measurement of change in perceived health status following foot surgery.

## Competing interests

There are no competing interests.

## Authors’ contributions

AJM undertook the data analysis and prepared the manuscript. TEK assisted in manuscript preparation and proof reading of all drafts. All authors read and approved the final manuscript.

## Supplementary Material

Additional file 1 EQ-5D. Click here for file

## Author details

^1^Department of Podiatric Surgery, County Health Partnerships, Park House Health and Social Care Centre, Burton Road, Carlton, Nottingham NG4 3DQ, UK. ^2^Department of Podiatric Surgery, Derbyshire Community Health Service, Ilkeston Community Hospital, Heanor Road, Ilkeston, Derbyshire DE7 8LN, UK.

## References

[B1] Maher AJ Kilmartin T Patient-reported outcomes: a new direction for podiatric surgery Podiatry Now 2010 13 10 36 37

[B2] Rudge G Tolafield D A critical assessment of a new evaluation tool for podiatric surgical outcome analysis British Journal of Podiatry 2003 6 4 100 110

[B3] Maher AJ Metcalfe SA A report of UK experience in 917 cases of day care foot surgery using a validated outcome tool Foot (Edinb) 2009 19 2 101 106 20307458 10.1016/j.foot.2009.01.002

[B4] Maher AJ Metcalfe SA First MTP joint arthrodesis for the treatment of hallux rigidus: results of 29 consecutive cases using the foot health status questionnaire validated measurement tool Foot 2008 18 3 123 130 10.1016/j.foot.2008.04.004 20307425

[B5] Spruce MC Bowling FL Metcalfe SA A longitudinal study of hallux valgus surgical outcomes using a validated patient centred outcome measure Foot (Edinb) 2011 21 3 133 137 21316938 10.1016/j.foot.2011.01.012

[B6] Claisse PJ Jones LA Mehata R Reporting foot surgery outcomes in everyday practice: Using a foot-related quality of life measure British Journal of Podiatry 2005 8 4 112 117

[B7] Maher AJ Kilmartin TE Patient Reported Outcomes following the combined rotation scarf and Akin's osteotomies in 71 consecutive cases Foot (Edinb) 2011 21 1 37 44 21146398 10.1016/j.foot.2010.11.002

[B8] Dawson J Coffey J Doll H Lavis G Cooke P Herron M Jenkinson C A patient-based questionnaire to assess outcomes of foot surgery: validation in the context of surgery for hallux valgus Qual Life Res 2006 15 7 1211 1222 10.1007/s11136-006-0061-5 17001437

[B9] Bowling A Bowling A, Ebrahim S Measuring health outcomes from the patient's perspective Handbook of Health Research Methods 2005 Open University Press, Maidenhead 428 444

[B10] Instrument selection criteria http://phi.uhce.ox.ac.uk/inst_selcrit. php#Respon

[B11] Kazis LE Anderson JJ Meenan RF Effect sizes for interpreting changes in health status Med Care 1989 27 3 Suppl S178 189 2646488 10.1097/00005650-198903001-00015

[B12] Dawson J Boller I Doll H Lavis G Sharp R Cooke P Jenkinson C The MOXFQ patient-reported questionnaire: assessment of data quality, reliability and validity in relation to foot and ankle surgery Foot (Edinb) 2011 21 2 92 102 21602039 10.1016/j.foot.2011.02.002

[B13] The Manchester-Oxford Foot Questionnaire PROs http://www.isis-innovation.com/outcomes/orthopaedic/modfq.html

[B14] Garrow AP Papageorgio AC Silman AJ Thomas E Jayson MI Macfarlane GJ Development and validation of a questionnaire to assess disabling foot pain Pain 2000 85 107 113 10.1016/S0304-3959(99)00263-8 10692609

[B15] Dawson J Doll H Coffey J Jenkinson C Responsiveness and minimally important change for the Manchester-Oxford foot questionnaire (MOXFQ) compared with AOFAS and SF-36 assessments following surgery for hallux valgus Osteoarthritis Cartilage 2007 15 8 918 931 10.1016/j.joca.2007.02.003 17383907

[B16] Harrison T Fawzy E Dinah F Palmer S Prospective assessment of dorsal cheilectomy for hallux rigidus using a patient-reported outcome score J Foot Ankle Surg 2010 49 3 232 237 10.1053/j.jfas.2010.02.004 20303801

[B17] Marinozzi A Martinelli N Panasci M Cancilleri F Franceschetti E Vincenzi B Di Martino A Denaro V Italian translation of the Manchester-Oxford Foot Questionnaire, with re-assessment of reliability and validity Qual Life Res 2009 18 7 923 927 10.1007/s11136-009-9508-9 19588271

[B18] Dawson J Boller I Doll H Lavis G Sharp R Cooke P Jenkinson C Responsiveness of the Manchester-Oxford Foot Questionnaire (MOXFQ) compared with AOFAS, SF-36 and EQ-5D assessments following foot or ankle surgery The Journal of Bone and Joint Surgery British Volume 2012 94 2 215 221 10.1302/0301-620X.94B2.27634 22323689

[B19] Kind P Dolan P Gudex C Williams A Variations in population health status: results from a United Kingdom national questionnaire survey British Medical Journal 1998 316 736 741 10.1136/bmj.316.7133.736 9529408 PMC28477

[B20] A new facility for the measurement of health-related quality of life. The EuroQol Group Health Policy 1990 16 3 199 208 10109801 10.1016/0168-8510(90)90421-9

[B21] Brazier J Jones N Kind P Testing the validity of the Euroqol and comparing it with the SF-36 health survey questionnaire Qual Life Res 1993 2 3 169 180 10.1007/BF00435221 8401453

[B22] van Agt HM Essink-Bot ML Krabbe PF Bonsel GJ Test-retest reliability of health state valuations collected with the EuroQol questionnaire Soc Sci Med 1994 39 11 1537 1544 10.1016/0277-9536(94)90005-1 7817218

[B23] Dolan P Gudex C Kind P Williams A The time trade-off method: results from a general population study Health Econ 1996 5 2 141 154 10.1002/(SICI)1099-1050(199603)5:2<141::AID-HEC189>3.0.CO;2-N 8733106

[B24] Rabin R de Charro F EQ-5D: a measure of health status from the EuroQol Group Ann Med 2001 33 5 337 343 10.3109/07853890109002087 11491192

[B25] Norman R Cronin P Viney R King M Street D Ratcliffe J International comparisons in valuing EQ-5D health states: a review and analysis Value Health 2009 12 8 1194 1200 10.1111/j.1524-4733.2009.00581.x 19695009

[B26] Weiss RJ Brostrom E Stark A Wick MC Wretenberg P Ankle/hindfoot arthrodesis in rheumatoid arthritis improves kinematics and kinetics of the knee and hip: a prospective gait analysis study Rheumatology (Oxford) 2007 46 6 1024 1028 10.1093/rheumatology/kem017 17409135

[B27] Saro C Andren B Wildemyr Z Fellander-Tsai L Outcome after distal metatarsal osteotomy for hallux valgus: a prospective randomized controlled trial of two methods Foot Ankle Int 2007 28 7 778 787 10.3113/FAI.2007.0778 17666169

[B28] Walters SJ Brazier JE Comparison of the minimally important difference for two health state utility measures: EQ-5D and SF-6D Qual Life Res 2005 14 6 1523 1532 10.1007/s11136-004-7713-0 16110932

[B29] Analyse-it for Microsoft Excel http://www.analyse-it.com/

[B30] Wilkinson AN Maher AJ Patient expectations of podiatric surgery in the United Kingdom J Foot Ankle Res 2011 4 1 27 10.1186/1757-1146-4-27 22145971 PMC3251527

[B31] Tai CC Ridgeway S Ramachandran M Ng VA Devic N Singh D Patient expectations for hallux valgus surgery J Orthop Surg (Hong Kong) 2008 16 1 91 95 18453668 10.1177/230949900801600121

[B32] Radl R Leithner A Zacherl M Lackner U Egger J Windhager R The influence of personality traits on the subjective outcome of operative hallux valgus correction Int Orthop 2004 28 5 303 306 10.1007/s00264-004-0574-x 15241625 PMC3456971

[B33] Schneider W Knahr K Surgery for hallux valgus. The expectations of patients and surgeons Int Orthop 2001 25 6 382 385 10.1007/s002640100289 11820447 PMC3620794

[B34] Mahomed NN Liang MH Cook EF Daltroy LH Fortin PR Fossel AH Katz JN The importance of patient expectations in predicting functional outcomes after total joint arthroplasty J Rheumatol 2002 29 6 1273 1279 12064846

[B35] Henn RF Kang L Tashjian RZ Green A Patients' preoperative expectations predict the outcome of rotator cuff repair J Bone Joint Surg Am 2007 89 9 1913 1919 10.2106/JBJS.F.00358 17768186

[B36] Dawson J Thorogood M Marks SA Juszczak E Dodd C Lavis G Fitzpatrick R The prevalence of foot problems in older women: a cause for concern J Public Health Med 2002 24 2 77 84 10.1093/pubmed/24.2.77 12141589

[B37] Macran S Kind P Collingwood J Hull R McDonald I Parkinson L Evaluating podiatry services: testing a treatment specific measure of health status Qual Life Res 2003 12 2 177 188 10.1023/A:1022257005017 12639064

[B38] Hurst NP Kind P Ruta D Hunter M Stubbings A Measuring health-related quality of life in rheumatoid arthritis: validity, responsiveness and reliability of EuroQol (EQ-5D) Br J Rheumatol 1997 36 5 551 559 10.1093/rheumatology/36.5.551 9189057

[B39] Brazier J Roberts J Tsuchiya A Busschbach J A comparison of the EQ-5D and SF-6D across seven patient groups Health Econ 2004 13 9 873 884 10.1002/hec.866 15362179

[B40] Pickard AS Johnson JA Feeny DH Shuaib A Carriere KC Nasser AM Agreement between patient and proxy assessments of health-related quality of life after stroke using the EQ-5D and Health Utilities Index Stroke 2004 35 2 607 612 10.1161/01.STR.0000110984.91157.BD 14726549

