# Supplementary material for: An analysis of Euroqol EQ-5D and Manchester Oxford Foot Questionnaire scores six months following podiatric surgery
Source: J Foot Ankle Res. 2012 Jul 9;5:17. doi: 10.1186/1757-1146-5-17 (PMC3489846; doi:10.1186/1757-1146-5-17)
Supplement: Additional file 1 — EQ-5D. [file 1757-1146-5-17-S1.pdf]

Health Questionnaire

English version for the UK (validated for Ireland)

SAMPLE

© 1990 EuroQol Group EQ-5D™ is a trade mark of the EuroQol Group

By placing a tick in one box in each group below, please indicate which statements best describe your own health state today.

Mobility

I have no problems in walking about ❑

I have some problems in walking about ❑

I am confined to bed ❑

Self-Care

I have no problems with self-care ❑

I have some problems washing or dressing myself ❑

I am unable to wash or dress myself ❑

Usual Activities (*e.g. work, study, housework, family or leisure activities)*

I have no problems with performing my usual activities ❑

I have some problems with performing my usual activities ❑

I am unable to perform my usual activities ❑

Pain/Discomfort

I have no pain or discomfort ❑

I have moderate pain or discomfort ❑

I have extreme pain or discomfort ❑

Anxiety/Depression

I am not anxious or depressed ❑

I am moderately anxious or depressed ❑

I am extremely anxious or depressed ❑

SAMPLE

© 1990 EuroQol Group EQ-5D™ is a trade mark of the EuroQol Group

To help people say how good or bad a health state is, we have drawn a scale (rather like a thermometer) on which the best state you can imagine is marked 100 and the worst state you can imagine is marked 0.

We would like you to indicate on this scale how good or bad your own health is today, in your opinion. Please do this by drawing a line from the box below to whichever point on the scale indicates how good or bad your health state is today.

Your own health state today

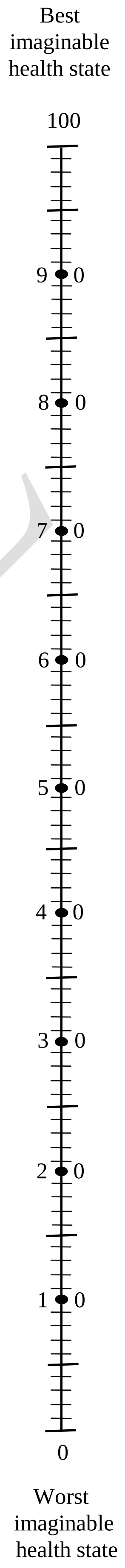

SAMPLE

© 1990 EuroQol Group EQ-5D™ is a trade mark of the EuroQol Group
